# Assessing spatial distribution, genetic variants, and virulence of pathogen *Mycoplasma agassizii* in threatened Mojave desert tortoises

**DOI:** 10.1002/ece3.10173

**Published:** 2023-06-04

**Authors:** Shalyn N. Bauschlicher, Chava L. Weitzman, Victoria Martinez, C. Richard Tracy, David Alvarez‐Ponce, Franziska C. Sandmeier

**Affiliations:** ^1^ Department of Biology Colorado State University – Pueblo Pueblo Colorado USA; ^2^ Research Institute for the Environment and Livelihoods Charles Darwin University Darwin Northwest Territory Australia; ^3^ Department of Biology University of Nevada Reno Nevada USA

**Keywords:** exo‐α‐sialidase, host–pathogen dynamics, mycoplasma, testudines, virulence gene, wildlife disease

## Abstract

Mojave desert tortoises (*Gopherus agassizii*), a threatened species under the US Endangered Species Act, are long‐lived reptiles that experience a chronic respiratory disease. The virulence of primary etiologic agent, *Mycoplasma agassizii*, remains poorly understood, but it exhibits temporal and geographic variability in causing disease outbreaks in host tortoises. Multiple attempts to culture and characterize the diversity of *M. agassizii* have had minimal success, even though this opportunistic pathogen chronically persists in nearly every population of Mojave desert tortoises. The current geographic range and the molecular mechanisms of virulence of the type‐strain, PS6^T^, are unknown, and the bacterium is thought to have low‐to‐moderate virulence. We designed a quantitative polymerase chain reaction (qPCR) targeting three putative virulence genes annotated on the PS6^T^ genome as exo‐α‐sialidases, enzymes which facilitate growth in many bacterial pathogens. We tested 140 *M. agassizii*‐positive DNA samples collected from 2010 to 2012 across the range of Mojave desert tortoises. We found evidence of multiple‐strain infections within hosts. We also found the prevalence of these sialidase‐encoding genes to be highest in tortoise populations surrounding southern Nevada, the area from which PS6^T^ was originally isolated. We found a general pattern of loss or reduced presence of sialidase among strains, even within a single host. However, in samples that were positive for any of the putative sialidase genes, one particular gene (528), was positively associated with bacterial loads of *M. agassizii* and may act as a growth factor for the bacterium. Our results suggest three evolutionary patterns: (1) high levels of variation, possibly due to neutral changes and chronic persistence, (2) a trade‐off between moderate virulence and transmission, and (3) selection against virulence in environmental conditions known to be physiologically stressful to the host. Our approach of quantifying genetic variation via qPCR represents a useful model of studying host–pathogen dynamics.

## Graphical abstract

We used qPCR of potential virulence genes and growth factors of the opportunistic pathogen, *Mycoplasma agassizii*, to survey effects on disease, disease severity, and microbial loads across the range of the Mojave desert tortoise. This study elucidates regional molecular mechanisms leading to disease in this enigmatic host–pathogen system.

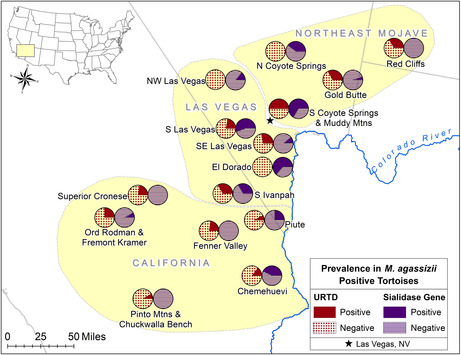

## 1 INTRODUCTION

Amid increasing urgency to develop standards in monitoring pathogen variation and movement across landscapes, wildlife biologists play an important role in developing pathogen‐specific methodology to understand ongoing threats of emerging diseases (Watsa and WDSFG, 2020). Many standard methods for surveillance of pathogen variation, including next‐generation sequencing (NGS), whole‐genome shotgun (WGS) sequencing, and sequencing of cultured isolates, are often biased in detecting the dominant or fastest‐growing strains while missing rare species or strains (Dyrhovden et al., 2019; Varghese et al., 2017). Alternatively, probe‐based quantitative PCR (qPCR) can be very specific, which makes it ideal for targeted pathogen surveys even if its specificity can result in false negatives due to slight changes in the target sequence (Braun et al., 2014; Geraci‐Yee et al., 2022; Rockett et al., 2011).

For *Mycoplasma agassizii*, the pathogen that causes upper respiratory tract disease (URTD) in Mojave desert tortoises (*Gopherus agassizii*; Brown et al., 1994), culture methods and antibody testing have had limited effectiveness in diagnostic testing and identifying strain variation (Braun et al., 2014; Luzuriaga‐Neira et al., 2021; Sandmeier, Weitzman, et al., 2017). In thirty years since its first isolation from its desert tortoise hosts, only two strains of *M. agassizii* have been isolated from this tortoise species (Brown et al., 1994, 2001; Jacobson et al., 1991). Hydrolysis probe‐based quantitative PCR has been instrumental in diagnosing *M. agassizii* in tortoises, as loads of *M. agassizii* DNA commonly are too low to sequence accurately (Braun et al., 2014). These assays have also led to insights on ecological patterns of disease (Aiello et al., 2014; Braun et al., 2014; Sandmeier et al., 2018). For example, low loads of *M. agassizii* often do not cause disease, and *M. agassizii* persists in tortoises for many years (Jacobson et al., 2014; Sandmeier et al., 2018; Sandmeier, Maloney, et al., 2017; Weitzman et al., 2017). Recrudescence of *M. agassizii* loads within hosts and transmission from other tortoises both are known to cause increases in disease prevalence in populations (Aiello et al., 2016; Sandmeier, Maloney, et al., 2017). However, the mechanisms causing this variation in growth is unknown and no clear virulence factors have been identified for this bacterium (Luzuriaga‐Neira et al., 2021; Sandmeier et al., 2018).

Virulence of *M. agassizii* is thought to be relatively low in desert tortoises in years in which environmental conditions do not deviate from mean conditions, outbreaks of disease are relatively rare, and levels of morbidity are low (Jacobson et al., 2014; Sandmeier et al., 2009; USFWS, 2015). Virulence is associated with adaptive antibody responses in the host, which appear to maintain local inflammation of the mucosal membranes (Sandmeier, Maloney, et al., 2017). Inflammation and nasal exudate seem to increase the chances of transmission among hosts, linking virulence and transmission (Alizon et al., 2008; Ebert & Bull, 2008). Importantly, innate immune mechanisms appear to be more effective at reducing loads of *M. agassizii* than adaptive immune mechanisms, which is common in reptiles (Sandmeier, Maloney, et al., 2017; Zimmerman et al., 2010). Success of new variants of *M. agassizii* likely is a balance between transmission and virulence, mediated by these host immune responses (Sandmeier & Tracy, 2014).

Recent genetic analysis of *M. agassizii* from wild tortoises found low amounts of genetic variation and confirmed that the type‐strain from the Mojave Desert, PS6^T^ (ATCC® 700616^T^), appears to have persisted in desert tortoise populations after its initial isolation in the early 1990s (Brown et al., 1994, 2001; Luzuriaga‐Neira et al., 2021; Weitzman et al., 2017). To understand virulence and genetic variation of PS6^T^, we identified virulence gene candidates to target with qPCR using data obtained by the recent sequencing and annotation of this strain (Alvarez‐Ponce et al., 2018). We targeted three annotated exo‐α‐sialidase genes found in PS6^T^, because sialidases are enzymes identified as important virulence factors in similar bacterial pathogens (Brown et al., 2004; Severi et al., 2007). Bacterial sialidases can function as virulence factors by freeing host monosaccharides called sialic acids, abundant throughout the mucosa of deuterostomes (Corfield, 1992; Lewis & Lewis, 2012). In doing so, they make additional nutritional sources available for bacterial growth, expose new sites for adhesion on host substrate, and increase biofilm formation (Hardy et al., 2017; Severi et al., 2007; Vimr et al., 2004). Therefore, we predicted that sialidases of *M. agassizii* increase bacterial growth rates and lead to increased disease (Michaels et al., 2019; Sandmeier et al., 2018; Weitzman et al., 2017).

The Mojave desert tortoise*–M. agassizii* system provides an opportunity to analyze low‐to‐moderate virulence under stabilized conditions, where a chronic, opportunistic pathogen evolved with slow immune responses and slow transmission rates of its host (Aiello et al., 2016; Luzuriaga‐Neira et al., 2021; Sandmeier et al., 2018). Table 1 outlines multiple, non‐mutually exclusive hypotheses of how virulence genes may evolve and affect tortoises and ecological patterns across the landscape (Dykhuizen & Kalia, 2008; Ebert & Bull, 2008). We made several predictions about how these patterns may manifest in desert tortoise populations (Table 1). We expected a trade‐off between virulence and transmission rates, virulence to be affected by host physiology and immunocompetence, and possible multiple‐strain infections (Table 1).

**TABLE 1:** Theoretical patterns of evolution of virulence, specific to the Mojave desert tortoise–*Mycoplasma agassizii* system.

Theoretical evolution of virulence	Expected patterns in individuals	Expected geographic patterns	Desert tortoise–*M. agassizii* predictions	References
1. Selective sweep of virulence genes	The presence of virulence genes should be strongly associated with disease	Regions should exist in which all samples are genetically identical and contain these virulence genes	Unlikely: Disease usually presents with low morbidity, low mortality, and chronic persistence	Ebert and Bull (2008), Sandmeier et al. (2018) and Sandmeier and Tracy (2014)
2. Moderate virulence selected for due to increased transmission rate	Virulence genes, or loads of the genes, are associated with URTD (visible nasal discharge)	These genes are expected to be present in populations with moderate‐low tortoise densities to increase transmission rates	Likely: *M. agassizii* is mainly spread by nose‐to‐nose contact, between animals with nasal discharge	Alizon et al. (2008) and Aiello et al. (2016)
3. Moderate virulence selected against in populations with less effective immunocompetence	Presence of genes are not associated with URTD, as lower immunocompetence can also result in higher virulence	Past outbreaks in regions which experience either severe drought or prolonged winters led to mortality events and selected against virulence genes	Likely: In the driest/hottest and coldest parts of the range, documented outbreaks of URTD have led to mortality events and/or high levels of morbidity	Ebert and Bull (2008), Sandmeier et al. (2009) and Sandmeier et al. (2013)
4. Genetic drift created high variability in chronic, low‐moderate virulence infections	Mixed‐strain infections may be common in individuals. Load of virulence genes may be more important than their presence/absence in causing URTD	No regional pattern	Likely: Recrudescence of URTD has been shown to be caused by persistence of *M. agassizii* across multiple years in tortoises. The majority of tortoises with *M. agassizii* harbor low‐level infections without any signs of URTD	Dykhuizen and Kalia (2008) and Sandmeier, Maloney, et al. (2017)
5. Putative virulence genes have evolved to avirulence	Neither presence nor loads of the genes are associated with URTD	No regional pattern	Unlikely: sialidase genes are often associated with virulence in mycoplasmas and other opportunistic pathogens	Brown et al. (2001) and Severi et al. (2007)

*Note*: We detail expectations for each pattern in individuals across the geographic landscape, and whether we deem these patterns to likely occur in our host–pathogen system.

**FIGURE 1:**
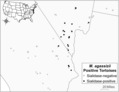
Distribution of *Mycoplasma agassizii*‐positive tortoise samples (*n* = 140) used in this study. Quantitative PCR identified 31 samples with at least one sialidase gene (black points), mainly clustered in southern Nevada, where PS6^T^ was originally isolated (Brown et al., 1994, 2001).

We used samples of *M. agassizii* collected via lavages the nasal cavities of wild tortoises to quantify genetic gains and losses of three putative exo‐sialidase genes, each of which is represented by one unique copy on the PS6^T^ genome (Alvarez‐Ponce et al., 2018). We used qPCR of the single‐copy 16S rRNA gene on the genome of all strains of *M. agassizii* to quantify total amounts of *M. agassizii* within samples (Braun et al., 2014; Weitzman et al., 2017). Samples were collected over a three‐year period (2010–2012) for health assessment of tortoise populations across the majority of the geographic extent of the Mojave Desert (Figure 1; described in Sandmeier et al., 2018; Weitzman et al., 2017). Throughout this article, we used “strain” interchangeably with genetic variant, to indicate quantitative differences of these virulence genes compared to PS6^T^ (Balmer & Tanner, 2011).

First, we confirmed that PS6^T^ sialidase genes were annotated correctly and likely to be functional by comparing them to genetic and amino acid sequences of known sialidases (Roggentin et al., 1993; Taylor, 1996; Vimr, 1994). We assessed how presence and loads of the three genes were associated and then tested four hypotheses. (1) We hypothesized that we would be able to detect strain variation of *M. agassizii* across the range of Mojave desert tortoises and within samples. If we found a partial loss of these genes within samples, this would indicate multiple‐strain infections in hosts. (2) We tested whether the presence of sialidase genes was associated with three measures of URTD: mycoplasmal load, signs of disease (nasal exudate), and disease severity (Weitzman et al., 2017; Sandmeier et al., 2018). We predicted that detection of the genes, as well as the number of the three genes detected in each sample (0–3), would predict greater *M. agassizii* loads, presence of disease, and disease severity (Roggentin et al., 1993; Taylor, 1996; Vimr, 1994). Because sialidase genes clustered on the landscape and were absent from many samples (Figure 1), we also analyzed the subsets of samples in which at least one putative sialidase gene was present. (3) We hypothesized that we would find a relationship between genetic loads of the sialidase genes when they were present and the three measures of URTD (*M. agassizii* load, disease presence, and disease severity). (4) Lastly, since Mojave desert tortoises are separated into three distinct genotypic populations driven by geographic distance, we hypothesized that prevalence of the sialidase genes would vary among these distinct host populations (Hagerty & Tracy, 2010; Shaffer et al., 2017).

## 2 METHODS

### 2.1 Study population and data collection

Across the entire Mojave Desert, 20 populations of wild tortoises (*n* = 419) were sampled during their active seasons (April–June) in 2010–2012 (described in Sandmeier et al., 2018; Weitzman et al., 2017). Individual tortoises were evaluated for presence (0 or 1) and severity (scored 0–6) of signs of disease (Sandmeier, Weitzman, et al., 2017; Weitzman et al., 2017). Nasal lavage samples were collected from each tortoise by rinsing 3 mL of sterile saline solution through the nares. The lavage samples were preserved in RNAlater stabilizing solution (Ambion Inc.) and placed on ice until they could be frozen within 12 h of collection (Weitzman et al., 2017). DNA was then extracted from 500 μL of the samples with the Qiagen DNeasy Blood and Tissue Kit (Qiagen Inc.) and stored at −20°C (Weitzman et al., 2017). One‐hundred forty samples from 17 local populations tested positive for *M. agassizii* by qPCR (Sandmeier, Weitzman, et al., 2017; Weitzman et al., 2017) and had enough volume of DNA sample remaining to be tested by four separate additional assays: three assays for each of the putative sialidase genes, and one assay for the quantification of *M. agassizii* (Braun et al., 2014).

### 2.2 Selection of sialidase genes

We used the annotated genome sequence of *M. agassizii* PS6^T^ (accession NQMN01; Alvarez‐Ponce et al., 2018) to identify three putative exo‐α‐sialidase genes (NCBI Prokaryotic Genome Annotation Pipeline 4.2; Tatusova et al., 2016). The three putative sialidase genes identified in PS6^T^ were located on two nodes of the genome, with accession numbers PAF55528.1 (“Gene 528”; Node 1; NQMN01000001.1), and PAF54905.1 and PAF54906.1, which appear physically next to each other, 513 bp apart, on the genome (“Genes 905 and 906”; Node 2; NQMN01000002.1).

We conducted BLASTp searches in GenBank to examine the accuracy of the annotation of the three genes by comparing sequence similarity to sialidase genes known to be functional in other bacteria. Using T‐Coffee (Di Tommaso et al., 2011), we aligned amino acid sequences of each *M. agassizii* PS6^T^ sialidase gene with other known bacterial sialidases to verify presence of conserved amino acid motifs, found in all functional bacterial sialidases: the YRIP motif (Arg‐Ile/Leu‐Pro) and the Asp‐box motif (Ser/Thr‐X‐Asp‐[X]‐Gly‐X‐Thr‐Trp/Phe) (Appendix S1; Roggentin et al., 1993; Taylor, 1996; Vimr, 1994).

### 2.3 Quantitative PCR assay design and optimization

Primers and probe candidates were generated with Primer Express Software v3.0.1 under default parameters for ideal qPCR chemistry (Thermo Fisher Scientific™). Target amplicons were verified to target CDS regions using NCBI GenBank, and BLASTn queries verified specificity for each Primer Express output. Specificity analyses using BLASTn eliminated any amplicon candidates with high similarity to non‐target sequences of other microbes. Each final amplicon with primer and probe sequences was selected to be specific to each PS6^T^ sialidase gene (Table 2; Appendix S2). The final selection of primer/probe sets was validated by in vitro specificity testing, with DNA of various microbes (*Pasteurella multocida* (ATCC® 43137); *Pasteurella canis* (ATCC® 43326); *Phocoenobacter uteri* (ATCC® 700972); *Gallibacterium anatis (*ATCC® 43329); *Escherichia coli* (ATCC® 87446); *Pasteurella testudinis* (ATCC® 33688); *Mycoplasma testudineum* (ATCC® 700618)) and the other target sialidase sequences. We also tested assays against one plate of *M. agassizii*‐negative samples that were collected from the same initial disease survey (Sandmeier et al., 2018; Weitzman et al., 2017).

**TABLE 2:** Quantitative PCR assay parameters for the three annotated *Mycoplasma agassizii* sialidase genes.

Primer target genes	Exo‐α‐sialidase
Accession number	PAF55528.1 (Gene 528)
Amplicon length	130 base pairs
Forward primer	5′‐CTAGTCAGGTTTTGTTTGCTCATAATG‐3′
Reverse primer	5′‐GGCGCATCAAGTTGACTATCTAAA‐3′
Hydrolysis probe	5′‐NED‐CACGCTTTACCGATACT‐MGBNFQ‐3′
Detection limit	50 copies
Optimal range	50–5,000,000 copies
Maximum intra‐assay CV	2.40%
Maximum inter‐assay CV	2.40%
Accession number	PAF54905.1 (Gene 905)
Amplicon length	98 base pairs
Forward Primer	5′‐TTGTCATCAGTTCTTCATGGTATTGA‐3′
Reverse Primer	5′‐CCTTCTCTTCTCCCACCCTGTT‐3′
Hydrolysis Probe	5′‐VIC‐CTTATTACTAAGTGGACCTTCA‐MGBNFQ‐3′
Detection limit	5 copies
Optimal range	50–5,000,000 copies
Maximum intra‐assay CV	2.22%
Maximum inter‐assay CV	2.22%
Accession number	PAF54906.1 (Gene 906)
Amplicon length	81 base pairs
Forward Primer	5′‐TTATCGCATGACACCACAAGGT‐3′
Reverse Primer	5′‐TTGCTTGCTGTTCAGTCTCCAT‐3′
Hydrolysis Probe	5′‐FAM‐AGGTGTTTACTTTATGACTAGAGAT‐MGBNFQ‐3′
Detection limit	5 copies
Optimal range	50–5,000,000 copies
Maximum intra‐assay CV	2.40%
Maximum inter‐assay CV	2.37%

*Note*: Detection limits are based on serial dilution curves of positive control standards from gBlocks and represent the lowest quantities of detection across plates with at least 95% certainty (Bustin et al., 2009).

Each qPCR was designed with the protocol used in Braun et al. (2014) to compare targeted sialidase gene copies to copies of *M. agassizii*. Each qPCR assay used a reaction volume of 12.5 μL TaqMan Environmental Master Mix 2.0 (Applied Biosystems), 0.2 μL forward primer (100 μM), 0.2 μL reverse primer (100 μM), 0.025 μL hydrolysis probe (100 μM), 2.5 μL sample DNA, and DNA‐free water to a final volume of 25 μL per well. Primers were optimized for an annealing temperature of 60°C. Mycoplasmal DNA from each uncultured tortoise lavage sample was assayed in triplicate in four separate assays, measuring pathogen load with 16S rRNA qPCR (Braun et al., 2014), as well as loads of each putative sialidase gene.

All Ct values were converted to the number of copies of the target DNA. We used synthetic gBlock gene fragments as amplicon‐specific positive controls (Table 2; Integrated DNA Technologies, San Diego, California, USA). The gBlock positive controls were run on each plate in triplicate in 10‐fold serial dilution curves, over a concentration range of 5 × 10^6^ copies to 5 copies along with negative controls.

### 2.4 Statistical analyses

**FIGURE 2:**
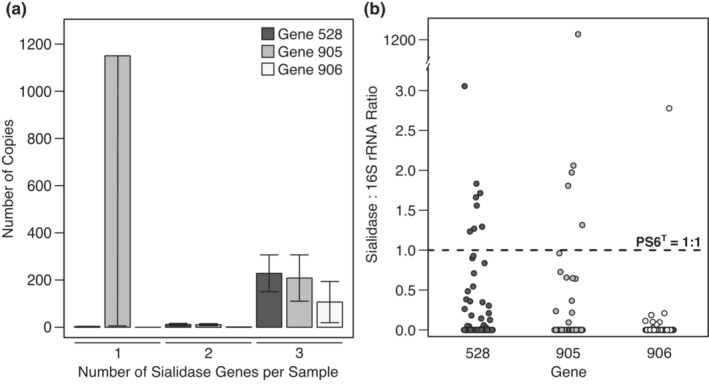
Genetic copies of the three putative sialidase genes. (a) Mean loads (±SE) of each of the genes in samples with 1, 2, or 3 of the genes present. The highest average loads were found in samples in which all three genes were detected. The load of gene 905 in the single‐gene sample is driven by a single data point. (b) Copies of sialidase genes per *M. agassizii* 16S rRNA gene. Each point represents calculated proportions of the sialidase gene copies to the copies of *M. agassizii* 16S rRNA gene, as compared to the expected 1:1 proportion (dotted line) observed on the annotated genome of PS6^T^. An overall genetic loss from the expected 1:1 proportion is visible.

We first tested whether the presence (0 = absent, 1 = present) of each of the three genes was correlated with each other via pairwise chi‐square analyses. Within the samples positive for any sialidase gene, we also used a correlation matrix to test for associations among loads of the three genes. We then tested for a relationship between total number of sialidase genes per sample (0–3) and mean load of each gene with a contingency analysis. These analyses revealed a high level of relationships among genes (Figure 2a), which informed our other analyses below. We plotted genetic ratios of copies of each putative sialidase gene to copies of 16S rRNA gene within each sample (Figure 2b; *n* = 140). Because each 16S rRNA gene is equivalent to one cell of *M. agassizii* (Alvarez‐Ponce et al., 2018; Braun et al., 2014), this visual analysis represents a loss or gain of sialidase genes per bacterial load within samples.

We used univariate regressions to test for associations between the presence of each gene and the load of *M. agassizii* (linear regression), presence of URTD (logistic regression), or severity of disease (ordinal logistic regression). In all analyses using load of *M. agassizii*, this variable was log‐transformed for normality. We used similar regression analyses to test for the effect of number of sialidase genes (0–3) on the load of *M. agassizii*, presence of URTD, or severity of disease. We then tested only the group of samples with sialidase genes (*n* = 31) and used multiple regression analyses to test whether loads of the three sialidase genes were associated with load of *M. agassizii* (linear regression), presence of URTD (logistic regression), or severity of disease (logistic regression). In these analyses, we focused on the influence of each gene (partial *p*‐value) in the presence of the other genes, since most genes occurred together in our samples (Figure 2a).

We used ESRI ArcMap™ (10.8.2) to spatially represent the presence and absence of the sialidase genes and URTD across the Mojave Desert. We used an ANOVA, with Tukey's post hoc comparisons, to test whether total genetic loads of sialidase genes varied among the three genetically distinct groups of Mojave desert tortoises: California, Las Vegas, and Northeast Mojave (Hagerty & Tracy, 2010; Shaffer et al., 2017).

## 3 RESULTS

### 3.1 Sialidase genes qPCR

Our standard nr database BLASTp searches found that gene 528 showed similarity (no E‐value cutoff) to 1087 sialidase protein sequences identified from respiratory bacteria. Significant similarities (E‐value cutoff 10^−20^) were found to seven other *Mycoplasma* spp. sialidase genes, including homologous similarity to a *Mycoplasma alligatoris nanI* sialidase gene (AAR98792.1), which codes a well‐studied virulence factor (BLASTp E‐value = 4 × 10^−83^, 100% query cover, 37% identities; Brown et al., 2004; Michaels et al., 2019). Gene 905 showed similarity to 76 bacterial sialidase protein sequences, with the 11 most significant similarities to *Mycoplasma* spp. sialidase genes. No homologs to gene 905 were found in the search, but significant similarity to the same *M. alligatoris nanI* sialidase gene (BLASTp E‐value = 10^−28^, 62% query cover, 27% identities) was observed. Gene 906 lies next to gene 905, and it exhibits a shorter gene sequence by 221 bp. Gene 906 showed similarity to six putative sialidase protein sequences within the *Mycoplasma* genus, including an annotated sialidase gene of *M. alligatoris* (EEF41391.1), but there is no reference to this gene in published virulence research of *M. alligatoris*, and the overall similarity was low (BLASTp E‐value 10^−17^, 67% query cover, 28% identities). No sialidase genes outside of the *M. agassizii* species showed highly significant similarity to gene 906 (E‐value cutoff 10^−20^). Genes 905 and 906 are highly similar in BLASTp search results (E‐value 6 × 10^−150^, 87% query cover, 48% identities). When the two genes were aligned using BLASTp, the results show even higher similarity (E‐value 8 × 10^−159^, 96% query cover, 46% identities; Appendix S1).

We located conserved amino acid motifs which are present on all functional bacterial sialidases: all three genes contain the YRIP motif, which occurs in the active site of bacterial sialidases (Roggentin et al., 1993). Genes 528 and 905 have one repeat of the Asp‐box motif, which likely functions in protein folding for the β‐propellor domain, an important structural element in bacterial sialidases (Appendix S1; Quistgaard & Thirup, 2009; Roggentin et al., 1993; Vimr, 1994). We detected genetic variations in the Asp‐box motif of gene 906. It is unclear whether these variations represent loss‐of‐function mutations, but because all functional bacterial sialidases contain both motifs, we were unable to verify likely functionality of gene 906 (Appendix S1; Quistgaard & Thirup, 2009; Roggentin et al., 1993; Vimr, 1994).

In silico and in vitro specificity tests verified that the qPCR assays amplified only the target genes from *M. agassizii*. The specific probe‐primer assays did not cross‐react with any other sialidase sequences and all DNA samples from *M. agassizii*‐negative tortoises remained negative for all three target genes. Table 2 reports average parameters for each of the three qPCR assays based on standard curves.

### 3.2 Statistical analyses

Out of these 140 *M. agassizii*‐positive samples, 28 were positive for gene 528, 14 were positive for gene 905, and eight were positive for gene 906 (Figures 1 and 2). Samples tended to have more than one sialidase gene, and we found that the presence of these genes correlated with each other significantly (Figure 2a; pairwise comparisons of 528 and 905: χ^2^ = 27.826, *p* < .0001; 528 and 906: χ^2^ = 29.459, *p* < .0001; 905 and 906: χ^2^ = 28.953, *p* < .0001). Loads of genes, however, were not correlated (*p >* .100 in all comparisons). The number of sialidase genes present in each sample (0–3) was positively associated with the total load of sialidase genes (*R* ^2^ = .52; *p* < .0001). Most samples showed multiple‐strain infections, with high levels of variation between the number of copies of the sialidase genes compared with the number of copies of *M. agassizii* (16S rRNA gene; Figure 2b). In some samples, sialidase genes were present in multiple copies compared to *M. agassizii* load, indicating gene duplication, but most samples contained fewer sialidase genes than mycoplasmal load (Figure 2b). In other words, samples positive for sialidase genes tended to also contain *M. agassizii* variants without sialidase genes.

**FIGURE 3:**
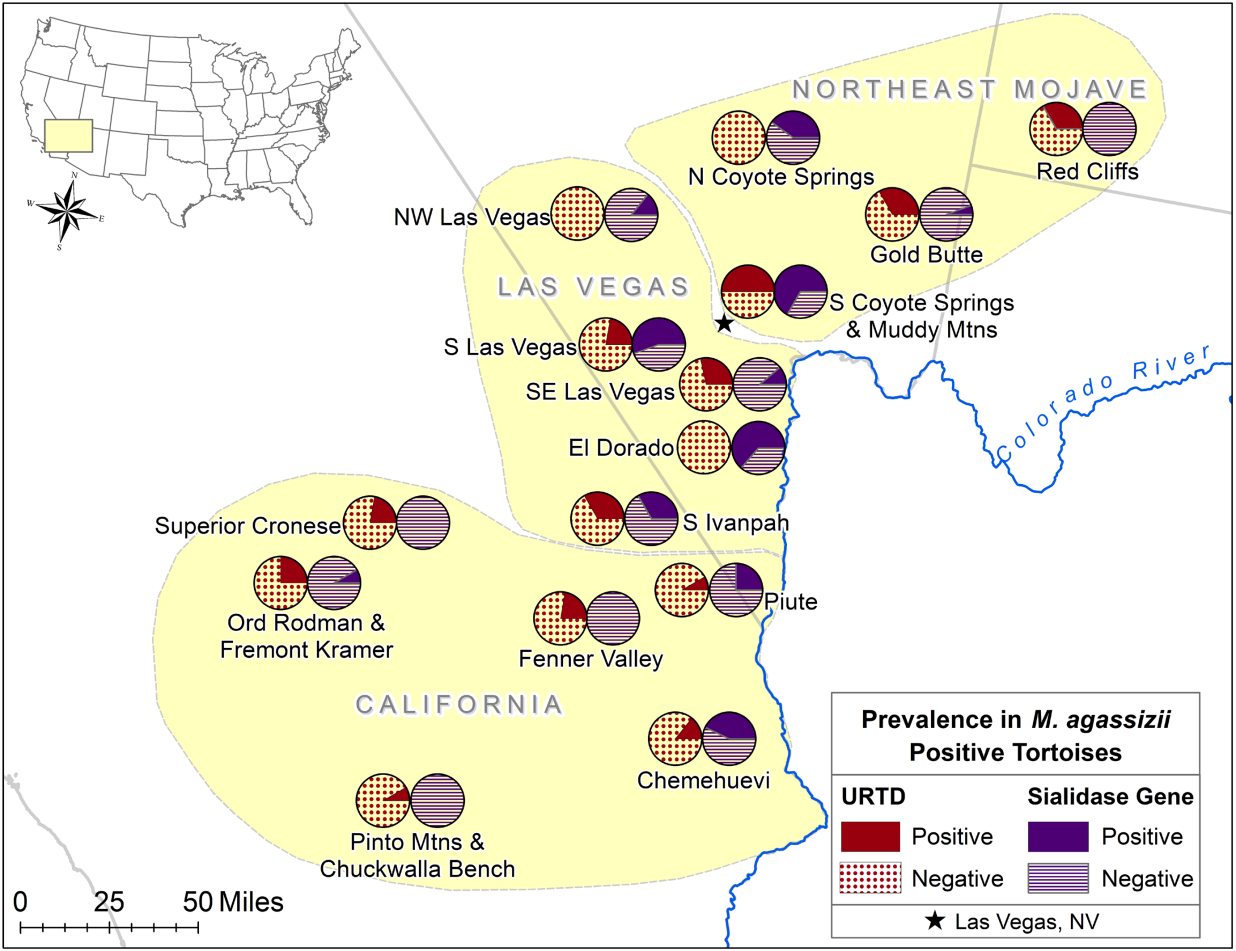
Prevalence of disease and prevalence of sialidase genes among population groups of Mojave desert tortoises (*n* ≥ 5 for labeled groups) within three distinct genetic populations (Hagerty & Tracy, 2010). Pie chart locations represent the approximate centroids of the sampled individuals in a valley. Visible clusters of sialidase genes do not always appear coincident with URTD. Solid color in the pie charts indicates the proportion positive for either measure.

In range‐wide, univariate analyses (*n* = 140), the presence of gene 906, but not 528 nor 905, was associated with load of *M. agassizii* (*R* ^2^ = .04; *p* = .023; *p* = .738; *p* = .056; respectively). The presence of genes 528, 905, and 906 were not associated with the presence of URTD (Figure 3; *p* = .647; *p* = .226; *p* = .571; respectively). The presence of genes 905 and 906, but not 528, were both associated with severity of disease (Uhler *R* ^2^ = .01, *p* = .044; Uhler *R* ^2^ = .03, *p* = .015; *p* = .642; respectively). The numbers of genes in each sample (0–3) was not associated with load of *M. agassizii* (*R* ^2^ = .05, *p* = .056), but was significantly associated with URTD (Uhler *R* ^2^ = .06, *p* = .039) and severity of disease (Uhler *R* ^2^ = .04, *p* = .020).

**FIGURE 4:**
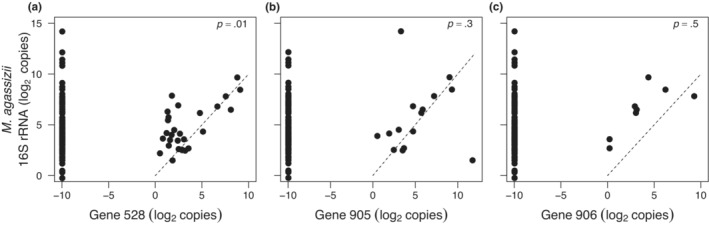
Associations between sialidase gene loads (*n* = 31) with loads of *M. agassizii* (*p* = .036, *R* ^2^ = .27). (a) gene 528, partial *p* = .012; (b) gene 905, partial *p* = .260; (c) gene 906, partial *p* = .541.

In analyses of sialidase‐positive samples (*n* = 31), only the load of 528 (partial *p* = .012), but not 905 (partial *p* = .260) or 906 (partial *p* = .541) was associated with the load of *M. agassizii* (Figure 4; whole model *R* ^2^ = .27, *p* = .036). The loads of all three genes (528: partial *p* = .034; 905: partial *p* = .003; 906: partial *p* = .007) were associated with the presence of URTD (whole model Uhler *R* ^2^ = .42, *p* = .005). Finally, loads of the three genes were not associated with disease severity (528: partial *p* = .627; 905: partial *p* = .150; 906: partial *p* = .110).

**FIGURE 5:**
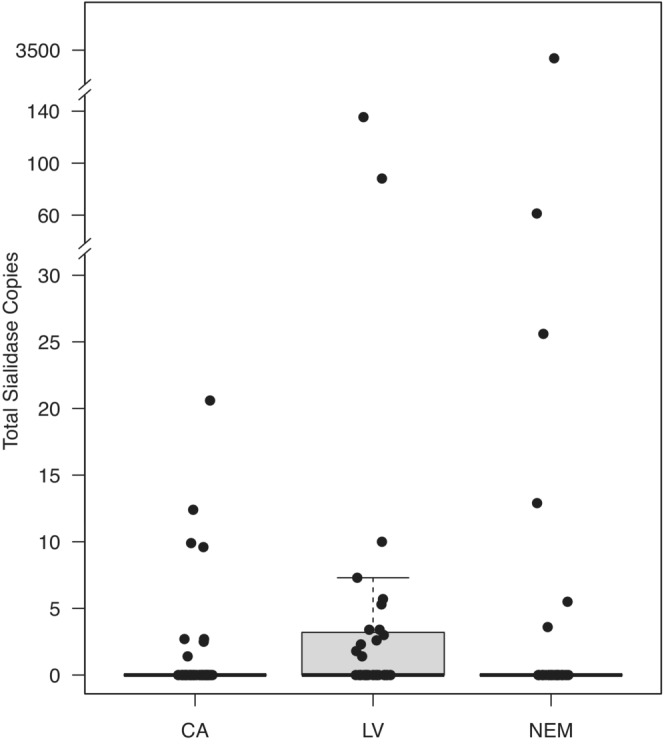
In genetic populations, the “Las Vegas” population had significantly higher average copies of sialidase than the “California” population (*p* = .007). Tortoise populations vary in sample size of *Mycoplasma agassizii*‐positive samples (California: *n* = 62; Las Vegas: *n* = 43; Northeast Mojave: *n* = 35).

Total load of sialidase genes significantly varied among the three genetic populations of tortoise hosts (*F*_2,137_ = 4.800, *p* = .010; Figure 5). There was no difference between the “Northeast Mojave” and the other two genetic populations (Las Vegas: *p* = .268; California: *p* = .438), but there was a significant higher level of sialidase genes in the “Las Vegas” versus the “California” populations (*p* = .007; Figure 5). Across the geographic range, sialidase genes were especially prevalent around the Las Vegas area and generally absent from southwestern California and the northeastern Mojave (Figures 1 and 3).

## 4 DISCUSSION

### 4.1 Summary of genetic variation within and across tortoise hosts

Specific to our hypotheses, we found that a variety of *M. agassizii* genetic strains occur across the Mojave as indicated by the absence of PS6^T^ sialidase genes in a majority of samples (Figure 2b). Similarly, we detected multiple‐strain infections within individual hosts (Figure 2b). We found mixed support for our hypotheses of a relationship between sialidase genes and virulence based on three measures of disease: mycoplasmal load, presence of disease, and severity of signs of disease. Low *R* ^2^ values suggest that the biological relevance of observed relationships is weak. However, in sialidase‐positive samples, load of gene 528 was strongly associated with higher loads of *M. agassizii* and loads of all three genes were strongly associated with increased presence of URTD, suggesting important within‐host dynamics involving growth rates and other mechanisms of virulence such as evasion of immune responses (Table 1). Spatially, we found an absence of these sialidase genes in the southwestern and northeastern extremes of the Mojave Desert and a distribution centered around southern Nevada (Figures 1 and 3). To summarize, the type‐strain PS6^T^ is not highly virulent (Sandmeier, Maloney, et al., 2017), but higher loads of all three putative sialidase genes are associated with an increased likelihood of mild signs of disease when they are present (Sandmeier et al., 2018; Weitzman et al., 2017).

Most samples with high loads of sialidase genes contained all three genes, suggesting a synergism in functionality (Figure 2a). Both genes 528 and 905 are likely functional sialidase genes based on in silico comparisons to known sialidase genes in other organisms. Of the three targeted PS6^T^ sialidase genes, 528 was present in the most samples and seems to play a role in bacterial nutrition, as its load was linked to higher loads of *M. agassizii* (Figure 4a), a predictor of recurrent URTD in tortoises and higher prevalence of disease in populations (Aiello et al., 2016; Sandmeier, Maloney, et al., 2017). Gene 528 is also a homolog of the *M. alligatoris nanI* protein, which increases mycoplasmal growth in highly virulent mycoplasmal infections in alligators (Michaels et al., 2019). Genes 905 and 906 were not associated with increased loads of *M. agassizii*, but both were associated with disease, suggesting other functions such as providing potential sites for adhesion on degraded mucosal substrate or avoiding immune detection (Corfield, 1992; Taylor, 1996; Vimr et al., 2004). When sialidase activity frees host molecules, this can allow bacteria to mimic host tissue and evade immune response by incorporating host sugars into their own membrane (Vimr, 1994).

Gene 906 may lack sialidase functionality based on in silico analyses and the current understanding of bacterial sialidase gene structure, and we do not view it as a functional gene (Appendix S1; Roggentin et al., 1993; Taylor, 1996; Vimr, 1994). In addition, it occurred only in eight samples that also contained genes 528 or 905, complicating the interpretation of its effect on disease.

### 4.2 Multiple‐strain infections

The multiple‐strain infections that we documented here could have occurred by direct transmission of multiple strains or from multiple transmission events, as *M. agassizii* is known to persist for years in healthy Mojave desert tortoises (Sandmeier et al., 2018; Sandmeier, Weitzman, et al., 2017). Alternatively, within‐host evolution could also lead to a diversity of strains (Table 1; Ebert & Bull, 2008; Sandmeier & Tracy, 2014). High levels of genetic diversity due to genetic drift, within and among individual hosts, can be a hallmark of chronic opportunistic pathogens which generally co‐exist with their hosts (Ebert & Bull, 2008). The long lifespan of tortoises, persistence and recrudescence of *M. agassizii*, and occasional transmission events, likely maintain low‐level, genetically diverse, multiple‐strain infections (Table 1, Figure 2b; Sandmeier et al., 2018).

There may be important ecological implications of multiple‐strain infections, largely based on effects of microbial interactions within the host, although effects on virulence are often unpredictable in such systems (Balmer & Tanner, 2011; Sofonea et al., 2017). The fact that many tortoises had lower levels of sialidase genes than *M. agassizii* suggests that even a small number of strains in a tortoise's respiratory tract can free nutrients, potentially benefiting other strains of *M. agassizii* or other bacterial species in the same location (Lewis & Lewis, 2012; Vimr, 1994).

### 4.3 Virulence‐transmission balance

We interpret our results as a possible balance between *M. agassizii* persistence in a host at low enough levels to evade innate immune responses and higher growth rates that cause inflammatory responses and higher transmission events (Table 1; Aiello et al., 2016). An interesting aspect of this host–pathogen system is that *M. agassizii* and URTD have been shown to be negatively associated with innate immunity—specifically, high levels of B1 lymphocytes which produce natural antibodies and phagocytize bacterial cells (Sandmeier et al., 2013, 2018; Slama et al., 2022). Low levels of *M. agassizii* likely persist in the host without innate response completely clearing the infection (Sandmeier et al., 2018; Slama et al., 2022). Conversely, high levels of inflammation and nasal exudate seem only temporarily effective in reducing URTD in desert tortoises and seem to maintain chronic disease and risk of transmission (Aiello et al., 2016; Sandmeier, Maloney, et al., 2017). The low levels of sialidase genes in many samples (Figure 2b) may represent a balance of virulence and transmission that primarily results in low‐level morbidity, but increased transmission rates. The duplication of sialidase genes in a handful of samples may imply the importance of these genes in transmission, as mycoplasmas have minimal genomes in which gene loss is more common than gene duplication (Razin, 1996; Razin & Hayflick, 2009).

Virulence genes may not exist in areas in which hosts experience conditions known to negatively impact tortoise physiology, such as drier and colder weather regimes, due to past host mortality events (Table 1; Figure 3). For example, tortoises in southern California historically experienced the most extreme drought events, the largest decreases in population densities, and the only well‐documented disease outbreaks associated with high mortality rates (USFWS, 2015). A highly virulent strain of *M. agassizii* was isolated from one population, “Fremont Kramer” (Figure 3), during a disease outbreak (Brown et al., 1994; Jacobson et al., 1991), and only low prevalence of the sialidase genes was detected in the same area during the 2010–2012 survey. Tortoises in the northernmost population in Utah experience a colder climate and hibernate communally in caves, factors thought to promote transmission of *M. agassizii* and disease (Sandmeier et al., 2013, 2018), and we detected no sialidase genes in this area (Figures 1 and 3). In both cases, more virulent strains may overwhelm individual tortoises, tipping virulence levels to higher host mortality rates, thus leading to local extirpation of virulent strains (Table 1).

This theoretical balance between virulence and transmission suggests that in the middle of the tortoises' range, where populations reach medium densities and experience less extremes of environmental conditions, a low‐to‐moderate virulence of *M. agassizii* is favored (Table 1; Berry & Murphy, 2019; Corn, 1994; Ebert & Bull, 2008).

### 4.4 Diagnostic considerations for quantifying *M. agassizii*

These hypotheses could be tested over time, especially if any outbreaks of disease are detected during routine desert tortoise monitoring activities (USFWS, 2015). Over recent years, large numbers of oral swab samples have been collected in and around the Las Vegas Valley for the US Fish and Wildlife Service due to tortoise translocations off of solar energy construction sites (Averill‐Murray, pers. comm.). Samples positive for *M. agassizii* could be tested for sialidase genes to confirm localized impacts of loads of these genes on disease (Figures 3 and 4). Assays for sialidase genes 528 and 905 could provide a tool for regional disease and health management in tortoise populations in regions of southern NV regions and eastern CA (Figure 3), especially in times of increased environmental stochasticity and sudden regional changes in disease prevalence (Averill‐Murray et al., 2021; Berry et al., 2020; Peterson, 1996).

This study demonstrated the strengths of using qPCR to study intraspecific microbial diversity in uncultured samples by targeting putative virulence genes and showed that qPCR can provide a sensitive technique to quantify gene‐specific variation across a host species' range (Brown et al., 2001; Hardy et al., 2017; Luzuriaga‐Neira et al., 2021). However, it is highly likely that our gene‐specific qPCRs did not detect other sialidase genes, as even small mutations affect detection and accuracy of highly specific qPCR assays (Braun et al., 2014; Rockett et al., 2011; Vanysacker et al., 2014). Developing similar assays for additional putative virulence genes of PS6^T^, and for other strains as they are sequenced, may provide an increasingly more complete picture of varying levels of disease risk across the landscape.

We suspect that difficulties culturing *M. agassizii* may be influenced by presence of sialidase. The two *M. agassizii* strains successfully cultured in the past, PS6^T^ (Brown et al., 2001; Jacobson et al., 1991) and an unnamed California strain no longer available to researchers (Brown et al., 1994), were grown in SP‐4 medium, which is rich in sialic acids due to compounds such as fetal bovine serum (Brown et al., 2001; Corfield, 1992; Jacobson et al., 1991; Sherblom et al., 1988). These previous studies tested several types of media, and growth success was only achieved using SP‐4 (Brown et al., 2001; Jacobson et al., 1991). Successful culturing of unknown strains which are clearly present in tortoises may require bound sialic acids within media to be broken down, as some strains may have the ability to catabolize sialic acid even without their own sialidase gene (Lewis & Lewis, 2012; Vimr, 1994). Perhaps supplementing sialidase enzyme in order to free up sialic acids bound in the culture medium may allow isolation of a greater diversity of strains.

## 5 CONCLUSION

A greater diversity of pathogen‐surveillance methods will increase knowledge of the mechanisms causing disease, both in threatened species such as Mojave desert tortoises, and other taxa of wildlife (Watsa and WDSFG, 2020). We demonstrated that qPCR methods may be adapted for tracking genetic variants, which may be a more accessible method to survey targeted genetic variation across a large geographic expanse and especially effective for identifying variation in bacteria which occur in multiple‐strain infections or are difficult to culture. We were also able to use this technique to suggest hypotheses for the evolution and current prevalence of virulent and avirulent strains. Our study design can easily be adapted for use in other host–pathogen systems to better understand functional dynamics, especially of chronic diseases with complex etiologies.

## AUTHOR CONTRIBUTIONS

**Shalyn N. Bauschlicher:** Data curation (lead); formal analysis (equal); investigation (lead); methodology (lead); project administration (equal); validation (equal); visualization (equal); writing – original draft (lead); writing – review and editing (equal). **Chava L. Weitzman:** Conceptualization (supporting); funding acquisition (equal); resources (supporting); validation (supporting); visualization (supporting); writing – review and editing (equal). **Victoria Martinez:** Methodology (supporting). **C. Richard Tracy:** Funding acquisition (equal); resources (equal); validation (supporting); writing – review and editing (equal). **David Alvarez‐Ponce:** Funding acquisition (equal); resources (equal); validation (supporting); writing – review and editing (equal). **Franziska C. Sandmeier:** Conceptualization (lead); data curation (supporting); formal analysis (equal); funding acquisition (equal); investigation (supporting); methodology (supporting); project administration (equal); resources (equal); software (lead); supervision (lead); validation (lead); visualization (equal); writing – original draft (supporting); writing – review and editing (equal).

## Supporting information


Appendix S1

Appendix S2

## Data Availability

Data are provided for peer review and are shared publicly in a repository: https://doi.org/10.6084/m9.figshare.20520771.v1.
