# Supplementary material for: Assessing spatial distribution, genetic variants, and virulence of pathogen Mycoplasma agassizii in threatened Mojave desert tortoises
Source: Ecol Evol. 2023 Jun 4;13(6):e10173. doi: 10.1002/ece3.10173 (PMC10239689; doi:10.1002/ece3.10173)
Supplement: Supplementary file 1 — Appendix S1 [file ECE3-13-e10173-s002.pdf]

Appendix I. Alignment of other *Mycoplasma* spp. and *Clostridium perfringens* sialidases with *M. agassizii* PS6^T sialidases. Location of the conserved YRIP and Asp-box sialidase motifs are annotated for the target genes only. Bold font indicates which gene the annotation corresponds to within the alignment. All three genes have only one repeat of each conserved motif.

```
Gene528            1 MKIQK-------------------------------------------------------
Gene905            1 MNKKKLI--------------LT-SF--IPL-IISSSVIAV-------------------
Gene906            1 MKVSKKF-------------IAISSLSLGAL-TLTATAIAC-------------------
M.testudinuem      1 MKLNDFE-------------I---------------------------------------
M.alligatoris      1 MNLKE-------------------------------------------------------
M.canis            1 MKKINKK-------------LAVYLLLSGAL-ITTTSLAIYFYANSQKIEKKKKSNSFSD
C.perfringens      1 MNYKGIT-----------------LILTAAM-VISGGNYVL-------------------
M.synoviae         1 MKKTKAQKEQQKKSQFKKRFWFTFTFLVGNLTIISSSLIPL-------------------
M.gallisepticum    1 MKKTKAQKDQQKKSQFKKRFWFTFTFLIGSITIISSFLIPL-------------------

Gene528            6 ------------------------------------------------------------
Gene905           24 ------------------------------------------------------ACST--
Gene906           28 ------------------------------------------------------TPST--
M.testudinuem      9 ------------------------------------------------------------
M.alligatoris      6 ------------------------------------------------------------
M.canis           47 SQINGFEFNIPEKHNTFVPNNNNHNDFIEQPEKSDVKFYNFQKKSDVDDSSFISLNFEG
C.perfringens     24 ------------------------------------------------------------
M.synoviae        42 ------------------------------------------------------SLKWIW
M.gallisepticum   42 ------------------------------------------------------SLKWIW

Gene528            6 ------------------------------------------------------------
Gene905           28 -----------------------------IIK----------------------------
Gene906           32 -----------------------------PPK----------------------------
M.testudinuem      9 --------LK----QNIKNLIDNDNF----------------------------------
M.alligatoris      6 ------------------------------------------------------------
M.canis          107 TKLLSESRLQIELEDNNKNLVVLDKF--KINNENKEVVFDTSDLTNNRKFNVKTITLNDQ
C.perfringens     24 ------------------------------------------------------------
M.synoviae        48 APLNIEEDLE----INIKDDLNGSRFISEINS----------------------------
M.gallisepticum   48 APLNIEEDLE----INIKDDLNGSRFISEINS----------------------------

Gene528            6 ------------------------------------------------------------
Gene905           31 ----DNTPLDEIKRFDLSKIP--SE------------------------------IIAKD
Gene906           35 ----EDPPTPPSKPT-PKPIPDPPK------------------------------GVDKE
M.testudinuem     23 ------------------------------------------------------------
M.alligatoris      6 ------------------------------------------------------------
M.canis          165 LIKNDNDIDDDTEKISFSTIP--SN-------TFIDFENSRIIESNENSATIEIELKTQD
C.perfringens     24 -----------------------------------------------------VKGSTLD
M.synoviae        76 ----DNLPE-------------FVNAFFNNKQYSIDDLNLKLVGVNYITGWVYVQASVKP
M.gallisepticum   76 ----DNLPE-------------FVNAFFNNKQYSIDDLNLKLAGVNYITGWVYVQASVKP

Gene528            6 ------------------------------------------------------------
Gene905           55 EY----------------------------------------------------------
Gene906           60 EY----------------------------------------------------------
M.testudinuem     23 ---------------SL-------------------------------------------
M.alligatoris      6 ------------------------------------------------------------
M.canis          216 NIKEISKS--AVSL-SFAYKNNNSKNY-LNITGILNKKQDKIFITANLNNLIHGESYFLE
C.perfringens     31 SGKNNSGY--EV------------------------------------------------
M.synoviae       119 NVKVAKKYQDAVKLFKLEFKNSQVKLFDPNV-----------------------------
M.gallisepticum  119 NVKVAKKYQDAVKLFKLEFKNSQVKLFDPNV-----------------------------

Gene528            6 ------------------------------------------------------------
Gene905           57 -------------------------------KE---LSKLKEKLESITADKPNQFFDLLD
Gene906           62 -------------------------------YK---------------------------
M.testudinuem     25 ------------------------------------------------------------
```

```
M.alligatoris        6 ------------------------------------------------------------
M.canis            272 KAKFISKPKGLFYNINNNYENAFYDFNKSNNKE--HEFVIHTDSETLFKNNSKFEFEVIG
C.perfringens       41 ---------------KVN---------NSE-SL--SSLGEYKDINLESSNASNITYDLGK
M.synoviae         150 -VTFLIKPE-----------------NNT--KEFTSEFQTREDLESFLNNADNISLSELQ
M.gallisepticum    150 -KTFLIKPE-----------------NNT--KEFTSEFQTREDLESFLNNADNISLSELQ

Gene528              6 ------------------------------------------------------------
Gene905             83 EKSKLEIE------NNLHEDFVISDFAYLENSSKNK--------TITVSLTFSNGETS--
Gene906             64 ----------------------IGWYDVHQN-----------------------------
M.testudinuem       25 ------------------------------------------------------------
M.alligatoris        6 ------------------------------------------------------------
M.canis            330 EMKNDPFENDQ-NEIKYHLSGVV----LDKRKIDFSKINENLKLKFSKVGSEETDVYASK
C.perfringens       74 -------Y------KNLDEGTIV----VRFNSK---------DSKIQSLLGISNSKTKNG
M.synoviae         190 DKLNLTFFDKNNVPLNLYTGAVV----TLSNPVKFTTASYVMDYKITWQIPVANDVFYGN
M.gallisepticum    190 DKLNLTFFDKNNVPLNLYTGAVV----TLSNPVKFTIASYVMDYKITWQIPAANDVFYGN

Gene528              6 ------------------------------------------------------------
Gene905            127 ------KIKRVEFKDLKVVLKEIPEVISPHILLNEVAYQWYLKNHKKVIQVQKSFENIS-
Gene906             74 ----------------------------------------------DKLSVADALKEKL-
M.testudinuem       25 -------KN-----KYS---------------------------------VYRNIENLIL
M.alligatoris        6 ------------------------------------------------------------
M.canis            385 ISYDP-NEN-----KLSFEIENSNSGDQF-----------ILKEIQVKNNETEQFENLD-
C.perfringens      108 YFNFYVTNS-----RVGFEL---------------------RNQKNEGNTQSGTENLI-
M.synoviae         246 LYSFS-LNG-----KLNLRLKEINSISSLYSDVDDIDFK--------KLQSKEIFKNLE-
M.gallisepticum    246 LYSFS-LNG-----KLNLWLKKINSISSLYSDVDDIDFK--------KLQSKEIFKNLE-

Gene528              6 ------------------------------------------------------------
Gene905            180 -VNDVIEISNNDLLEPFEIPESFELIIQVINDSKLKIQGSLEAQLILKSGDT-YFDDSYQ
Gene906             87 -PTEITSLKNSDLKEPLKGLEGYNVNLQIIKDLASNHEGTLFVKVVLEKDGKFYGEDGKS
M.testudinuem       40 THYKEHNDFLETLENFINLREE--L----KAGLKILINNLQILG-ID---CK--------
M.alligatoris        6 ------------------------------------------------------------
M.canis            427 -IT------NVD------------------KKLIIEYPISKSLE-V-DLINS-RSWESSL
C.perfringens      140 HMYKDVALNEDDNTVA--L-----KVE-KNKGYKLFLNGKMIKE-VKDTNTK-FLNNIEN
M.synoviae         291 -EVQNFSTSIETLDQFYNVPDD--LVVK-GQDFKLRYYVNKTAQ-IFDDVES-FLDNSNL
M.gallisepticum    291 -EVQNFSTSIETLDQFYNVPDD--LVVK-GQDFKLGYYVNKTAQ-TFDNVES-FLDNSNL

Gene528              6 ------------------------------------------------------------
Gene905            238 IY------------SRLKDVKIGSILVTGF-----------------------KKED---
Gene906            146 V-------------DKVDDAKTFRTKVSGL-----------------------KVRN---
M.testudinuem       82 -RSNIGIVLELKTYIDEIK---SE------------------------------------
M.alligatoris        6 ------------------------------------------------------------
M.canis            459 YPSYVFINL---KLKNNWNDEKIEKLLDQLT------KDDYSESK--------KNSIKNV
C.perfringens      190 LDSAFIGKT---NRYGQSNQYNFKGNIGFMNIYSEPLGDDYLLSKTGETKAKDEILVEGA
M.synoviae         345 ISSGYLTRM---TYDDKYKVVISKPNLDTFTKASSETQRKYLQSF-KQNKAPVKVTI---
M.gallisepticum    345 ISSGYLTRM---TYDDKHKVVISKPNLDTFTKASSETQRKYLQSF-KQNKAPVKVTI---

Gene528              6 ------------------------------------------------------------
Gene905            260 ------------------------------------------------------------
Gene906            167 ------------------------------------------------------------
M.testudinuem      102 -----QLIFFDIYINKGHTVN---------------------------------------
M.alligatoris        6 ------------------------------------------------------------
M.canis            502 LANIIYDNYKKIVKNNNVDIDNKIIILRLSDPADDSNFYSQDNNIFYFLLNETNKHFKNT
C.perfringens      247 VK----------------------------------------------------------
M.synoviae         398 ------------------------------------------------------------
M.gallisepticum    398 ------------------------------------------------------------

Gene528              6 --------------------------------------------ISSQVLF---------
Gene905            260 --------------------------------DKLKQENVYVQQLFKQFAF---------
Gene906            167 --------------------------------ISLADQKVTVEQVYKKFGF---------
M.testudinuem      118 --------------------------------KDISNLGIEISELEENIIF---------
```

```
M.alligatoris     6 --------------------------------------------LFNINLF---------
M.canis         562 KDIDEKWDIVNNSNTISISMIKNPFISNNRNGTAIENDHFKIKRLSSETLF---------
C.perfringens   249 --------------------------------------------TEPVDLF---------
M.synoviae      398 --------------------------------ANTVD--RTVEVFYTDFSFNKSPLVNGK
M.gallisepticum 398 --------------------------------ANTVD--RTVEVFYTDFSFNKSPLVNGK

                                          |YRIP|
Gene528          13 -------------AHNEANSHSYRIPSLLKLKNNDLIAIVDQRLDSQLDAPYSEINQVVK
Gene905         279 -------------QHKENQKHTYRIPSIAKY-QDKLYLQIDGRIDNKEDTPLNRIVQSLK
Gene906         186 -------------THNLNHKHSYRIPSIGKY-NGKLYLQIDGRIDNQSDAPFNRINQSIR
M.testudinuem   137 -------------KKDENFKGGFRIPSMSLLKDGQILFNCDRRFLNNLDAPYTNIDQVYK
M.alligatoris    13 -------------KANYQNSHSYRIPTLLKLKSGKLIAIVDQRLNSQLDAPYSSINQVIR
M.canis         613 -------------AHNEDGSHSYRIPNVTKLKNGKILSVVDKRVENISDY-NNSISQVFK
C.perfringens   256 -------------HPGFLNSSNYRIPALFKTKEGTLIASIDARRQGGADAPNNDIDTAVR
M.synoviae      424 YLFFNMSPPGDKFVYYTKDVNSWRIPGVIKTHDNKLIFNADKRVNNKDDR--GHLEQDMR
M.gallisepticum 424 YLFFNMSPPGDKFVYYTKDVNTWRIPGVVKTHDNKLIFNADKRVNNKDDR--GHLEQDIR

Gene528          60 VSKDD-QNFSPLKIIMSFEHNK-N---IK-ASFIDSSIIQ----D-----KFNRVHLIVD
Gene905         325 TFDLKTQQFSNAKDILSVRTDT-K---ER-ITLIDSNLTI----D----EENDIAHLIVD
Gene906         232 TYDLKTKTMSEMKDLITVKSNT-K---EK-IALIDSSFTI----D----DQKHIAHLIVD
M.testudinuem   184 LKNNNGK-ISDLKTFIKINSYS-D---DKPQSIIDLSLTN----E-----VDGYIYFMAD
M.alligatoris    60 ESFDNGRTWSEAKIILKLEKFS-T---AK-ASAIDSVTVQ----D----IKNNYLYLAVD
M.canis         659 ETLDGGKTWSQNKEILKIAVPK-K--NNR-GIAIDGIITEIEYFDEETQTNKTKLHFIVD
C.perfringens   303 RSEDGGKTWDEGQIIMDY-----P---DK-SSVIDTTLIQ----D----DETGRIFLLVT
M.synoviae      482 VSEDGGKTWSNPQTIVRISAKNTKDGANK-GQVIDGTMLE----V---FDKSINKHKLLY
M.gallisepticum 482 VSEDGGKTWSNPQTIVRISAKNTKDGANK-GQVIDGTMLE----V---FDKSINKHKLLY

Gene528         105 IFPSNG-GLMP-IVNK-LNPLNNLD-KVDQ-SGLPYLYDSNL--NKYY----LVLD--YK
Gene905         372 AFPSDA-GYIG-MSKSTFNQDMNLS-GIYN-SGNLKLLDRVK--DEFI-----EFK--IS
Gene906         279 AMPSEA-GVSG-RGRNAFINNLSLS-ALYN-SGNLNLVDRVK--DVML-----QFN--IQ
M.testudinuem   230 FFPGGA-GIFS-KTLNYFVTSENQNFGFY--KEYLIVEDLEN--ENYI-----FMKMVSS
M.alligatoris   107 IFPGGS-GLMP-LCKE-LNPLCNSDLGYTK-DKYLKLYDLYN--NNYI-----LR-----
M.canis         715 IFPGTNTGVPH-LSSG--NPWF----YIGD-QGYLKMWTKLNNRNNFDSRSSVLK---RV
C.perfringens   346 HFPSKY-GFWN-AGLG--SGFK----NIDG-KEYLCLYDSSG--KEFT-----VR-----
M.synoviae      534 VFGVSS-YLHTVHHERTIRDDGNTS-GLVNNNKWMLFIDKENQNQKFV-----AKP-ITV
M.gallisepticum 534 VFGVSS-YLHTVHDEWTIRGDGNTS-GLVNNNKWMLFIDKENLKQKFV-----AKP-ITV

Gene528         152 KTNEYFIFDQQNIK------------------VEKFKIKYYFDSSNSKFYFNLYELKNQK
Gene905         419 ERN-SNWYQGYNYKTK---------------EKTNVVIN--LEVKNNKFIFDVYEMKNAN
Gene906         326 DLS-KKWYQAYNEKTK---------------EKVNVVLH--LDQVGNDFILDIYEMKDAT
M.testudinuem   279 QKG-HFTYQGFTDKNKK-INFQNLNSEKLNLIKINLFLIFQFDNIESKFKGNFYIKNDSN
M.alligatoris   151 ETTQKFIYQAFLLKDK---NFNN--TNESNLVATKIYVDKSFNSKTSLLKGSVYENIDKL
M.canis         764 EGR-GNWFRRYILPAG--VSFNNNFTASTQLEETNTYVDMNYHQDTKSISGRVYENVMES
C.perfringens   385 EN---VVYDKDGNKTE---------------YTTNALGD-LF--KNGTKIDNINSS----
M.synoviae      586 DGD-SNWFKVFKVKNSSGVQWKD-IDDKTQLDEANIIIDNSL--KNGTITGNVYDNVPSN
M.gallisepticum 586 DGD-SNWFKVFKVKNSSRVQWKD-IDDTTQLDEANIIIDNSL--KNGTITGNVYDKVPSN

Gene528         194 -----N-----EYLYSLF-DATK---------N---------DRFHLTSGS---------
Gene905         461 S--NLNNVNEMKLIQSAF-ELFK--------NYENGKYE--RSKYKVIPQT---------
Gene906         368 K--DLSDKDNLTYVQSAM-EIFK--------DHDKGDFS--ASKYKLVTQV---------
M.testudinuem   337 E---FIDL--N---ISFF-SGA---------NFSKND-S--GLRYRALSTN---------
M.alligatoris   206 -----EDLNNKKPICSIW-DGFE---------NSSKEYK--KPKYFIDSTA---------
M.canis         821 DFDDPAALDSKKTEHSVF-DEPRKVTNVNNNTFEPLRNE--HAVYALAINS---------
C.perfringens   420 -----------T------------------------------APLKAKGTS---------
M.synoviae      642 I-------------FSTFGEPGR--------NSRSSEISRYISKYSIWDQRWPNDFGRNI
M.gallisepticum 642 I-------------FSTFGEPGR--------NSRSSEISEYISKYSIWDQRWPNYFVFNR

                                         |Asp-box|
Gene528         216 ------------------YLAHFISEDFGNSFELNSLLNYLFINKS-NNINATVVSPGKA
Gene905         499 ------------------FLSYLTIDLKTNEITFHRFLNDDLIK-E-KRTSIDLVSPGNG
Gene906         406 ------------------FLSYFTIDLKTDKVTFEGFLNDKLNK-T-NPTAIDLVGPGNG
M.testudinuem   367 ------------------YLSFFKFNIQTSQIEFICFINPWLENYKNIGANLVLNGPGKA
```

```
M.alligatoris     240 ------------------YIQLFVSKDDGLSWELINDITAQIRTKN-SKMNCLVLGPGQG
M.canis           869 ------------------HLATLESYDEGRTWTNLQWIDEKLSKHR-NNHKFVGTGVGNG
C.perfringens     430 ------------------YINLVYSDDDGKTWSEPQNINFQVK--K-DWMKFLGIAPGRG
M.synoviae        681 MQKNIYFANSRYYFDNILWTFQVESIDGGKTWTNLRNISPFLNR---KDEKWYINGVGNG
M.gallisepticum   681 MQKNAYFANSRYYFDNILWTFQVESIDGGKTWTNLRNVSPFLNR---KDQRWYINGVGNG

                                                   |Asp-box|
Gene528           257 VSIEDE---EKNEYLIFNVYETKNAM----PHKVYELKYNTGINKWTVGSYLNENLC---
Gene905           539 IILRHQKNAEDNGKLVFANYRINVLR----RSLGAYFMMKDRHGEWTHSNFIAGG-----
Gene906           446 IILRNQHNEADNGKLIFTNYRMTPQG----RHVGVYFMTRDKDGDWTASKFVA-------
M.testudinuem     409 LKLKYQ-NGNSKNRLIFKAYFTNDAS----SLGAITIYSDDYGNTWQTSEFIGGP-----
M.alligatoris     281 LYLQNQINKYISNKIIFPFYEVNYAF----PFHVFVSSSDDNGMNWTNSTYLNSKTN---
M.canis           910 IQLKHQANASINGRVIIPMYSMNNND----HY-MFFIYSDDKGKTWTKYTPTGFK-----
C.perfringens     469 IQIKNG---EHKGRIVVPVYYTNEKG----KQSSAVIYSDDNGKNWTIGESPNDNRKLEN
M.synoviae        738 IQLNHQRNSNGDVIAVFPFYLADQIDSRKWLERSKLMATRDGGKTWYEFADYPFEL----
M.gallisepticum   738 IQLNHQRNSNGDVIAVFPFYFANKRK----LERSKLMATKDGGKTWYELADYPFKL----

                                                              |Asp-box|
Gene528           307 ----------N--QWLSETAIIKSKDKTLYAISRNPNY-----GKLLVSKKKYHSNYWSD
Gene905           590 ------------ESNFTETTIVEQQDGTLVFIARNTM------NKLAIGYSYDGGKTFTN
Gene906           495 ------------TNSYSETSVVEQQDGTLVFIARNSL------GKLAIGYSYDGG-----
M.testudinuem     459 ------------KSRITEFDVTELQNGVLVAILRRMGS-----NTLSLSKSIDGGKTWNS
M.alligatoris     334 ----------KNWFWTSETQLIELSNGKILAFLRNPNQ-----DHLCISQSIDGGISWSA
M.canis           960 -------------TNLSESSFVETEDGTLYWFARHTGSFGQNTFRTFISKSTDGGMTWSS
C.perfringens     522 GKIINSKTLSDDAPQLTECQVVEMPNGQLKLFMRNLS------GYLNIATSFDGGATWDE
M.synoviae        794 -----------LPPGASESSISEDSKGNLWWLARRSDG-----RVFTLIKSEDGGKNWKV
M.gallisepticum   790 -----------LPPGASESSISEDSKGNLWWLARRSDG-----RVFTLIKSEDGGKNWKV

Gene528           350 LDYLKCNQS--QFD--LKNINTQIMHGVTNF-NYLND-EYILISLPST-QDRKEGNLFVF
Gene905           632 KAGNKDKMD--LYS--NVDLLSSVIHGIEYF-KYKGD-DYILLSGPSKQGGRREGKLFLV
Gene906               ------------------------------------------------------------
M.testudinuem     502 ISGNTDEVG--ISSIILNEYNSSVLQGIANF-TYLNS-HYLLITS-GS-NGRNEGKIWMI
M.alligatoris     379 LENNNESLS--LYK--IEPLNSQIMHGISTF-SHNNI-DYVLLSLPTT-KDRKNGKIFVF
M.canis          1007 PDNDASRKGKDMQI--GNPYDGNIFSGISYF-KWKNK-DYFLFSLSKS-VVRRNGSLFIA
C.perfringens     576 TVEKDTNV--------LEPYC--QLSVINYSQKIDGK-DAVIFSNPNA-NSRSNGTVRIG
M.synoviae        838 IFNDKRGI----FT--------RQFIGSTIF-NIKALGEKLIFSA----RNNGGGKIYLV
M.gallisepticum   834 IFNAKRGI----FT--------GQFIGSTIF-NIKALGEKLIFSA----SDNRGGKIYLV

Gene528           403 KNY---DFKNILLHHKIT-KE------DESFGYSNIEILNIENNKIKFAILYEMSYSKKI
Gene905           686 KNN---DFKNAKEIYRFN-RK------NDKFEYSNLELIDVDDEGVKIATLYETQTDENK
Gene906               ------------------------------------------------------------
M.testudinuem     556 KDF---ELNSITEIYSLN-S--------QTFGYSLIDVIKIEQNLFMINVVYEQNFSN--
M.alligatoris     432 DAN---DFNKPLAKFDVD-KD------NYSFGYSTIAILEKNDKKITFALLYECSQIKNA
M.canis          1062 DAT----FENIVELFRYDDNQ------REHFAYSYALVTNKTENYIDFISIYEASERFKI
C.perfringens     624 LINQVGTYENGEPKYEFD-WKYNKLVKPGYYAYSCLTEL--SNG--NIGLLYEGTPSEEM
M.synoviae        881 DIN---NPTTLEPIYTIN-R--------GSYGYSTTVTVNENDNYFDVLTFYEVDSPQK-
M.gallisepticum   877 DIN---NPRTLEPIYTIN-R--------GSYGYSTTVTFNENDNYFDVLTFYEVNSPQK-

Gene528           453 GLEWLGEEGQDETDYKNPSEIIYDVFELI--F
Gene905           736 --------------NKDVVEIVLEILKVKIY-
Gene906               --------------------------------
M.testudinuem     602 ------------------GELIYKKIRLKF-K
M.alligatoris     482 GLDWVGFNSDTNTDKGLASEILYKVFELT--L
M.canis          1112 ----ID-GGFDN-SRPQGGEIQLDKFRLWIKD
C.perfringens     679 ----------------SYIEMNLKYLQSGANK
M.synoviae        928 -----------------GKDINLKRLRVFVVS
M.gallisepticum   924 -----------------GRDINLKRLRVFVVS
```
