# Supplementary material for: Assessing spatial distribution, genetic variants, and virulence of pathogen Mycoplasma agassizii in threatened Mojave desert tortoises
Source: Ecol Evol. 2023 Jun 4;13(6):e10173. doi: 10.1002/ece3.10173 (PMC10239689; doi:10.1002/ece3.10173)
Supplement: Supplementary file 2 — Appendix S2 [file ECE3-13-e10173-s001.pdf]

**Appendix II.** Alignment of the qPCR primers and probe on each of the three *M. agassizii* PS6$^T$ sialidase gene target sequences. Primer sequences are highlighted in gold and the hydrolysis probe sequences are highlighted in blue.

Gene 528 Target Sequence

CTAGTCAGGTTTTGTTTGCTCATAATGAAGCAAATTCCCATTCATATCGCATTCCTTC
ACTATTAAAATTAAAAAATAATGATTTAATTGCTATAGTCGATCAACGTTTAGATAG
TCAACTTGATGCGCC

Gene 905 Target Sequence

TTGTCATCAGTTCTTCATGGTATTGAATATTTTAAATATAAAGGTGATGATTACTTAT
TACTAAGTGGACCTTCAAAACAGGGTGGGAGAAGAGAAGG

Gene 906 Target Sequence

TTATCGCATGACACCACAAGGTAGACATGTAGGTGTTTACTTTATGACTAGAGATAA
AGATGGAGACTGAACAGCAAGCAA
